# Persistent COVID-19 parosmia and olfactory loss post olfactory training: randomized clinical trial comparing central and peripheral-acting therapeutics

**DOI:** 10.1007/s00405-024-08548-6

**Published:** 2024-03-16

**Authors:** Elena Cantone, Luca D’Ascanio, Pietro De Luca, Dalila Roccamatisi, Ignazio La La Mantia, Michael J. Brenner, Arianna Di Stadio

**Affiliations:** 1 Department of Otolaryngology, Federico II University of Naples, Naples, Italy; 2 Department of Otolaryngology, Ospedali Riuniti Marche Nord, Fano, Italy; 3 Department of Otolaryngology, Fatebenefratelli Isola Tiberina-Gemelli Isola, Rome, Italy; 4 Psychology Department, UTIU, Rome, Italy; 5 GF Ingrassia Department, University of Catania, Catania, Italy; 6 Department of Otolaryngology-Head and Neck Surgery, University of Michigan Medical School, Ann Arbor, MI USA; 7 IRCCS Santa Lucia, Rome, Italy

**Keywords:** Anosmia, Parosmia, Coronavirus long-COVID, Palmitoylethanolamide, Alpha lipoic acid

## Abstract

**Purpose:**

Although COVID-19 anosmia is often transient, patients with persistent olfactory dysfunction (pOD) can experience refractory parosmia and diminished smell. This study evaluated four putative therapies for parosmia in patients with chronic COVID-19 olfactory impairment.

**Methods:**

After screening nasal endoscopy, 85 patients (49 female, 58%) with pOD and treatment-refractory parosmia were randomized to: (1) ultramicronized palmitoylethanolamide and luteolin + olfactory training (OT) (*umPEALUT group*, n = 17), (2) alpha-lipoic acid + OT (*ALA group*, n = 21), (3) umPEALUT + ALA + OT (*combination group*, n = 28), or 4) olfactory training (OT) alone (*control group*, n = 23). Olfactory function was assessed at baseline (T_0_) and 6 months (T_1_) using a parosmia questionnaire and Sniffin’ Sticks test of odor threshold, detection, and identification (TDI). Analyses included one-way ANOVA for numeric data and Chi-Square analyses for nominal data on parosmia.

**Results:**

The umPEALUT group had the largest improvement in TDI scores (21.8 ± 9.4 to 29.7 ± 7.5) followed by the combination group (19.6 ± 6.29 to 27.5 ± 2.7), both p < 0.01. The control and ALA groups had no significant change. Patients in the combination and umPEALUT groups had significantly improved TDI scores compared to ALA and control groups (p < 0.001). Rates of parosmia resolution after 6 months were reported at 96% for combination, 65% for control, 53% for umPEALUT and 29% for ALA (p < 0.001). All treatment regimens were well-tolerated.

**Conclusions:**

umPEALUT and OT, with or without ALA, was associated with improvement in TDI scores and parosmia, whereas OT alone or OT with ALA were associated with little benefit.

## Introduction

Qualitative disorders of smell, which include both distortions of perception in the presence of odorous stimuli (parosmia) and perception of an odor in absence of a physical stimulus (phantosmia) [1], are common and can be even more distressing for patients than loss of smell [2]. Whereas literature on post-COVD-19 olfactory dysfunction has emphasized quantitative disorders of smell (e.g., anosmia, hyposmia), parosmia remains understudied and has few evidence-based treatments. Often a transient phenomenon, parosmia can herald the recovery of lost smell; however, studies in the COVID-19 era have shown that parosmia can persist for years accompanying persistent loss of smell [3]. Although olfactory training (OT) can support recovery from post-viral smell loss, its efficacy for persistent parosmia has not been shown [4]; this finding might reflect damage of the olfactory neuroepithelium and bulb induced by SARS-CoV-2 [5]. Parosmia is thought to arise from altered signaling, neuronal loss, and aberrant connectivity of olfactory receptor neurons with the olfactory bulb [6, 7]. Neuroinflammation involving the olfactory bulb and higher brain centers may contribute to parosmia and poor recovery [8, 9].

These insights into the pathogenesis of parosmia and chronic olfactory dysfunction have provided an impetus for novel therapies aiming to reduce neuroinflammation and thereby support recovery. Combining ultramicronized palmitoylethanolamide and luteolin (umPEALUT) supplements with OT promoted recovery of quantitative smell loss from COVID-19 in randomized trials [10–12]; however, its effect on parosmia was less clear [13]. In the pre-COVID era, studies of alpha-lipoic acid (ALA) suggested potential benefit for post-viral loss of smell and parosmia, attributed to its anti-inflammatory and neuroprotective effects [14, 15]. We investigated whether treatment regimens involving umPEALUT, ALA, or both, combined with OT could ameliorate parosmia or quantitative smell dysfunction in patients with chronic olfactory dysfunction after SARS-CoV-2 infection.

## Materials and methods

Patients with known history of COVID-19 and anosmia/hyposmia with parosmia were recruited from the Otolaryngology Unit of a tertial referral center (University Hospital Federico II of Naples) from January 2022 to December 2022. All participating patients had been previously treated with olfactory training without resolution of their olfactory complaints. The study was conducted in accordance with Declaration of Helsinki standards for human studies and approved by the Institutional Review Board with number 93/19. All patients provided written consent to participate in the study and to have their data included in analyses and scientific reporting. The study had a single coordinator (ADS) and site leaders at the two participating centers (EC and LDA), each of whom were otorhinolaryngologists with a decade or more of expertise in evaluating quantitative and qualitative olfactory dysfunction.

### Inclusion criteria

Eligible patients were ages 18 to 60 years with a prior diagnosis of COVID-19 (positive nasopharyngeal swab for SARS-CoV-2 infection), smell disturbances resulting from the COVID-19 episode, no previous history of smell or tase alterations before COVID-19, and persistent olfactory dysfunction (smell loss and parosmia) at time of recruitment.

### Exclusion criteria

Exclusion criteria included known neuroinflammatory or neurodegenerative diseases, stroke or head trauma within the past 5 years, prior or ongoing treatment with chemo-radiotherapy, history or presence at time of the enrollment of sinonasal cancer or chronic rhinosinusitis, nasal allergy, presence of nasal polyps, other anatomical conditions that reduce nasal airflow including severe nasal fracture or severe nasal septal deviation (Class III unilateral deviation in contact with the lateral nasal wall with complaint of severe nasal obstruction, per Cottle Classification [16]), patients receiving drugs known to interact with olfactory function, and patients with severe psychiatric disorders.

Participants underwent nasal endoscopy to exclude nasal pathology and a structured medical history at T_0_ (baseline) and after 180 days of treatment (T_1_) was completed. Immediately after the nasal endoscopy and before being tested for olfactory function, a blinded randomization was performed in which participants were randomly assigned to one of four groups by computer. All participants in the study received daily OT [2, 4]. Treatment groups were as follows:

1. *umPEALUT group* in which patients consumed 1 sachet daily of ultramicronized Palmitoylethanolamide and Luteolin (umPEALUT) (Glialia 700 + 70; Epitech) plus daily OT;
2. *ALA group*, in which patients received 800 mg daily of alpha-lipoic acid (ALA) plus daily OT;
3. *Combined group*, in which patients consumed 1 sachet daily of umPEALUT and 600 mg of ALA plus daily OT; and
4. Control group, where patients received daily OT alone [2, 4].

Simple randomization was performed by assigning a consecutive number to each patient and allocating them to control group, ALA group, umPEALUT group, or combined group (ALA + umPEALUT) before knowing the results of the Threshold (T) Detection (D) and Identification (I) score (blinded randomization). The otolaryngologist who performed the TDI evaluation was blinded regarding the treatment used by the patient. Following assignment, participants underwent the Sniffin’ Sticks test battery by clinicians who were blinded to treatment assignments. Assessment included odor threshold (T), odor discrimination (D), and odor identification (I) subtests, with results summed to calculate a composite TDI-score [11, 17]. The T score can range from 1 to 16, D score from 0 to 16, and I score from 0 to 16, with normal function corresponding to higher scores (14–16) [11, 17]. Recovery was considered significant when a minimum of 5 points were recovered from T_0_ to T_1_ [17]. Patients received regular follow-up from the study team via phone call, electronic communication, or during routine office visits to verify adherence with the study regimen.

Following explanations to patients regarding qualitative disorders of smell, patients were queried about the presence or absence of parosmia or phantosmia, as previously described [18]. Additional follow-up questions were adapted from the questionnaire instrument described by Landis et. al. [19], which was simplified to dichotomous yes/no responses to mitigate stress and survey fatigue [18, 20]. Data on age, sex, and comorbidities were also collected. In addition, the duration of persistent olfactory disorder was recorded in months; this value was calculated based on the time elapsed from post-COVID negative nasopharyngeal swab for the SARS-CoV-2 virus to initial olfactory evaluation.

The primary outcome was resolution of parosmia; the secondary outcome was change in TDI score.

### Sample size calculation

The sample size was calculated as described by Wang and Ji [21], utilizing the protocol specific for Randomized Clinical Trials available at calculator.net (https://www.calculator.net/sample-size-calculator.html?type=1&cl=95&ci=6.5&pp=50&ps=&x=43&y=8), with design incorporating a 95% confidence interval and < 7% margin of error.

### Statistical analysis

To compare odor threshold, discrimination, identification, and composite TDI scores, Analysis of Variance (ANOVA) for repeated measures was performed, using values at baseline (T_0_) and 6-month (T_1_) time points as within-subject factor, and treatment groups were analyzed as between subject factors with Bonferroni-Holmes post hoc tests. Chi-Square tests (χ) were used to analyze the differences in prevalence of parosmia resolution between the four study groups. Multivariate analyses were performed analyzing the relationship between baseline TDI scores and parosmia; small sample size precluded adding covariates of sex and age to the multivariate model. Comparisons were considered significant at p < 0.05.

## Results

**Fig. 1:**
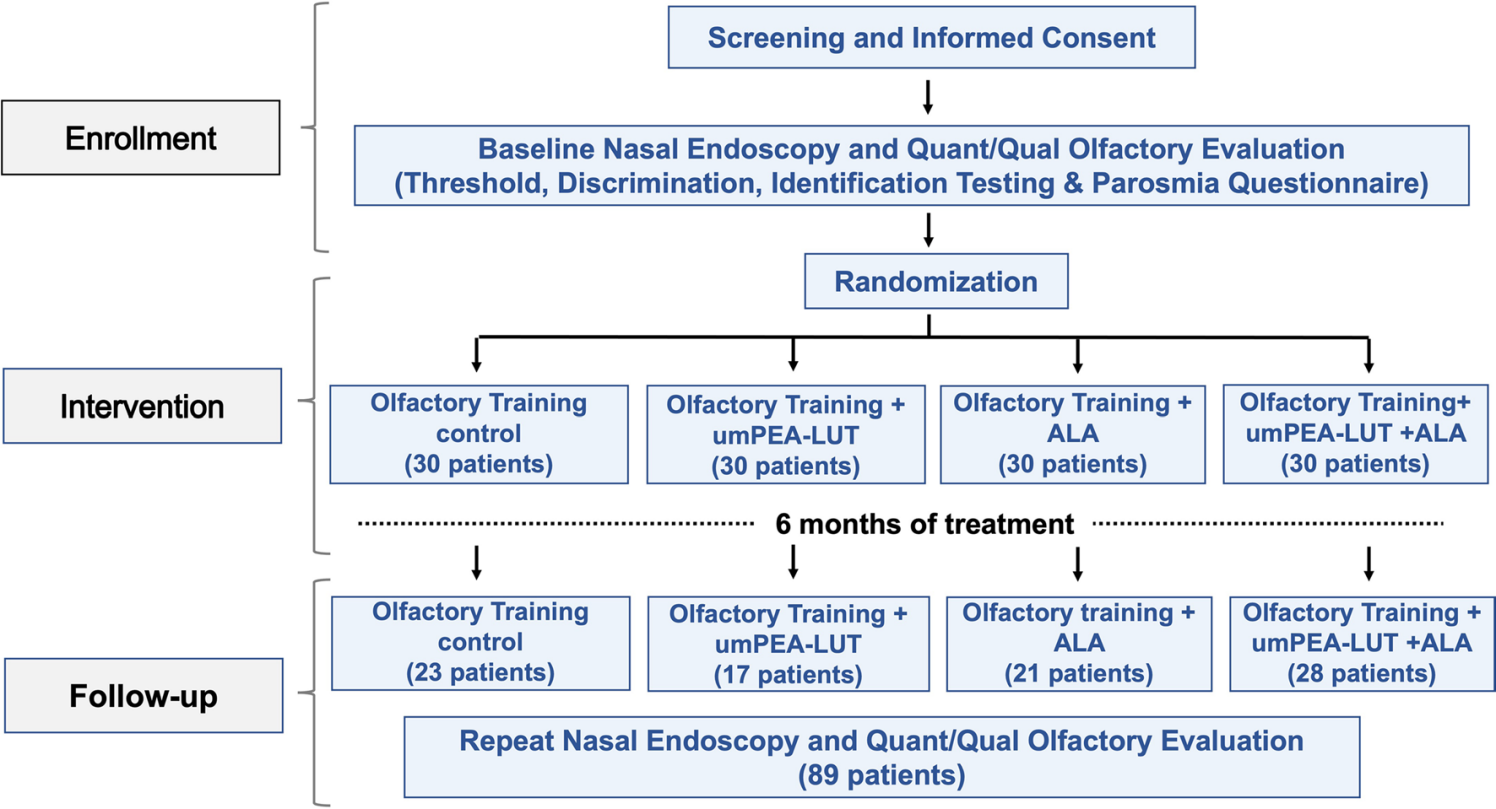
Figure plot shows the steps of the study, including patients’ allocation to the different groups at the enrollment and the final number after attrition, which included only those patients who completed three months of treatment and included in the data analyses

From the original 120 patients (30 patients for each group) enrolled, 31 were lost during follow-up or opted to discontinue participation during the 6-month study period (29.6% attrition rate), leaving 89 patients available for analysis. Analyzed study participants included 51 females and 38 males, with average age 43.4 ± 12.3 years. Of the 31 participants lost to follow-up, 7 were in the control group, 13 in the umPEALUT, 9 in the ALA, and 2 in the combination group. Despite the high attrition rate (> 25%), loss of patients did not create statistically significant differences in baseline characteristics in the final sample. Analyzed patients included 23 patients (13 women and 10 men, average age 52.1 ± 11.8 years) in the control group, 17 in the umPEALUT group (11 women and 6 men, average age 44.8 ± 12.2 years), 21 in the ALA group (9 women and 12 men, average age 35.9 ± 12.3), and 28 (18 women and 10 men, average age 42 ± 10.3 years) in the combination group (Fig. 1).

**Fig. 2:**
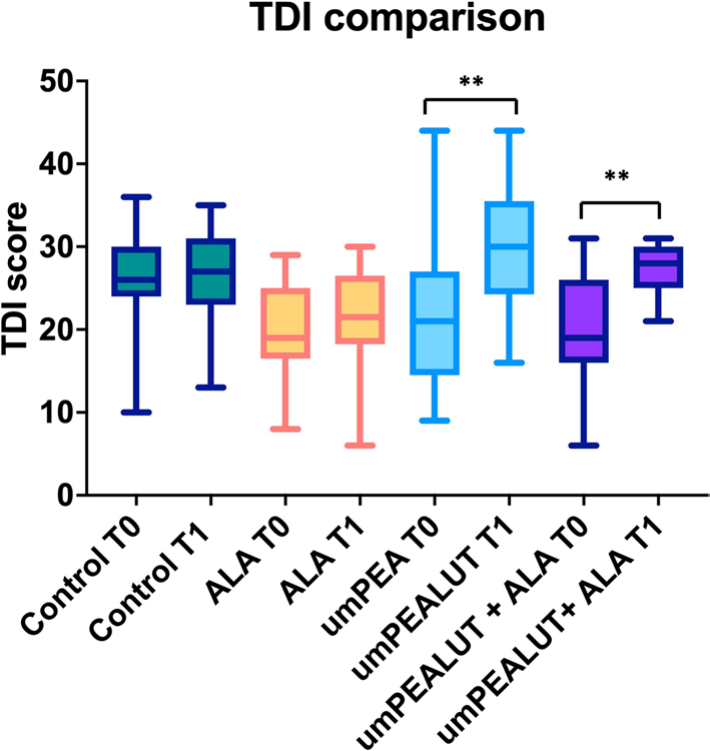
Comparison of the TDI between the four analyzed groups before (T_0_) and after 6 months of treatment (T_1_). A statistically significant improvement (**p < 0.01) was observed in the patients treated with OT and umPEALUT

Analyses of variance within groups were statistically significantly different (ANOVA: p < 0.0001). The analysis of each single group showed no statistically significant difference in the TDI scores of the control group before (T_0_) (average 26.9 ± 5.3) and after treatment (T_1_) (average 27.7 ± 5; BH: p > 0.05). Patients in the ALA group also did not show significant change in mean TDI scores (BH: p > 0.05) from T_0_ (average 19.3 ± 5.6) to T_1_ (average 21.7 ± 4.3). In contrast, patients in the umPEALUT group and the combination group showed a significant improvement of TDI scores from T_0_ to T_1_. For the umPEALUT group, mean TDI average scores were 18.6 ± 10.4 at the baseline (T_0_) and 29.7 ± 7.5 at T1 (BH: p < 0.01) (Fig. 2).

**Fig. 3:**
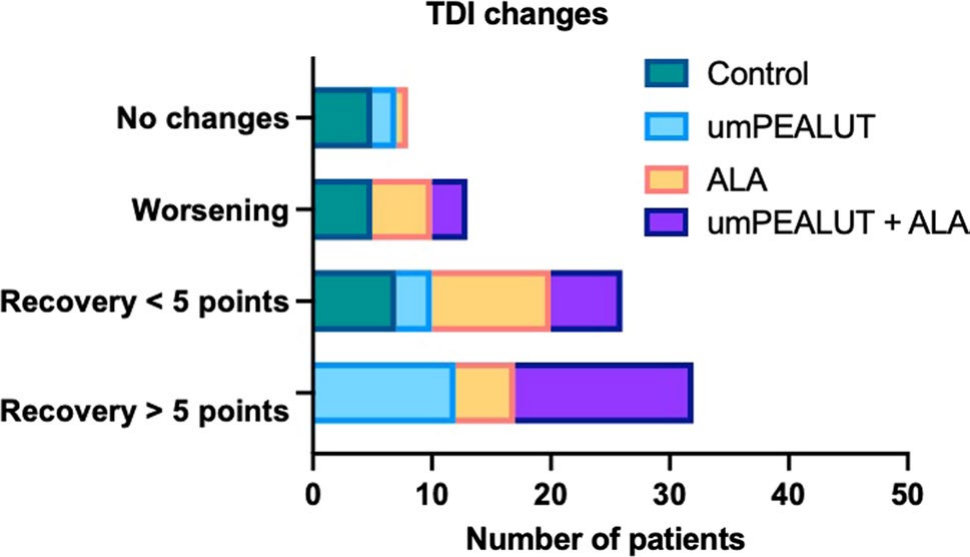
Details about the TDI recovery among the 4 groups. The higher recovery (> 5 points) was observed in the patients who used umPEALUT. The combination of umPEALUT plus ALA resulted in lower TDI scores than umPEALUT without ALA in some patients

For the combination group, mean TDI scores were 19.5 ± 6.3 at baseline and 27.5 ± 2.5 at T_1_ (BH: p < 0.01) (Fig. 2). Across groups a recovery over 5 points was observed in 64.7% (11 subjects) of patients in the umPEALUT group (average 10.4 ± 3.6); in 62.5% (15 patients) in the combination group (average 11.7 ± 3.4); and in 23.8% (5 cases) in the ALA group (average 5 ± 0). None of the patients in the control group recovered at least 5 points in TDI scores (Fig. 3).

The comparison between groups at the baseline (T_0_) showed statistically significant differences between control and umPEALUT (BH: p < 0.05), control and ALA (BH: p < 0.01), control and umPEALUT + ALA (BH: p < 0.01). No other statistically significant differences were observed at T_0_ between umPEALUT, ALA and unPEALUT + ALA groups. The comparison at T_1_ showed statistically significant differences (ANOVA: p < 0.0001), including lower TDI scores of the control versus the umPEALUT (BH: p < 0.01) as well as lower TDI scores of the ALA group versus umPEALUT (BH: p < 0.01) and combination (BH: p < 0.01). However, no statistically significant differences were observed between control and combination group at T_1_ (BH: p > 0.05); in fact, although the combined group patients had recovery of the olfactory functions compared to the baseline, their scores were the same as the control that started with higher score at T_0_ (Table 1). No statistically significant differences were identified between the control and the ALA group (BH: p > 0.05) or between umPEALUT and combination groups at T_1_, (both BH p > 0.05) (Fig. 2).

**Table 1:** Summary of the demographic characteristics of the groups and findings on quantitative and qualitative olfactory dysfunction at baseline (T_0_) and 180 days (T_1_)

Variable	Control group (n = 23) (Placebo + OT)	umPEA-LUT group (n = 17) (umPEA-LUT + OT)	ALA group (n = 21) (ALA + OT)	Combined group (n = 28) (umPEA-LUT + ALA and OT)
Mean age in years ± SD (CI95%)	52.1 ± 11.8 (25–62)	44.8 ± 11.81 (19–62)	35.9 ± 12.3 (25–64)	42 ± 10.29 (27–57)
Gender—n (%)				
Female	13 (57%)	11 (65%)	9 (43%)	18 (64%)
Male	10 (43%)	6 (35%)	12 (57%)	10 (36%)
Prevalence of parosmia, n (%)	23 (100%)	17(100%)	21 (100%)	24 (86%)
Months of olfactory loss, mean ± SD (range)	8.1 ± 1.8 (5–12)	10 ± 4.4 (4–20)	10.2 ± 4.13 (3–20)	15.3 ± 6.6 (7–24)
Baseline TDI (T_0_), mean ± SD (range)	26.9 ± 5.3 (17–36)	18.6 ± 10.45 (10–39)	19.3 ± 5.6 (8–29)	19.6 ± 6.29 (6–31)
Endpoint TDI (T_1_), mean ± SD (range)	27.7 ± 5 (19–35)	29.7 ± 7.5 (16–39)	21.7 ± 4.3 (13–30)	27.5 ± 2.5 (21–31)
Resolution of parosmia (n, %)	15 (65%)	9 (53%)	6 (29%)	23 (96%)

Multivariate analyses showed a weak association between TDI scores at the baseline or T2 and parosmia in the control group; a similar weak association was observed for the umPEALUT group, while a moderate association of TDI at T0 and T2 was observed both for ALA and umPEALUT + ALA groups.

**Fig. 4:**
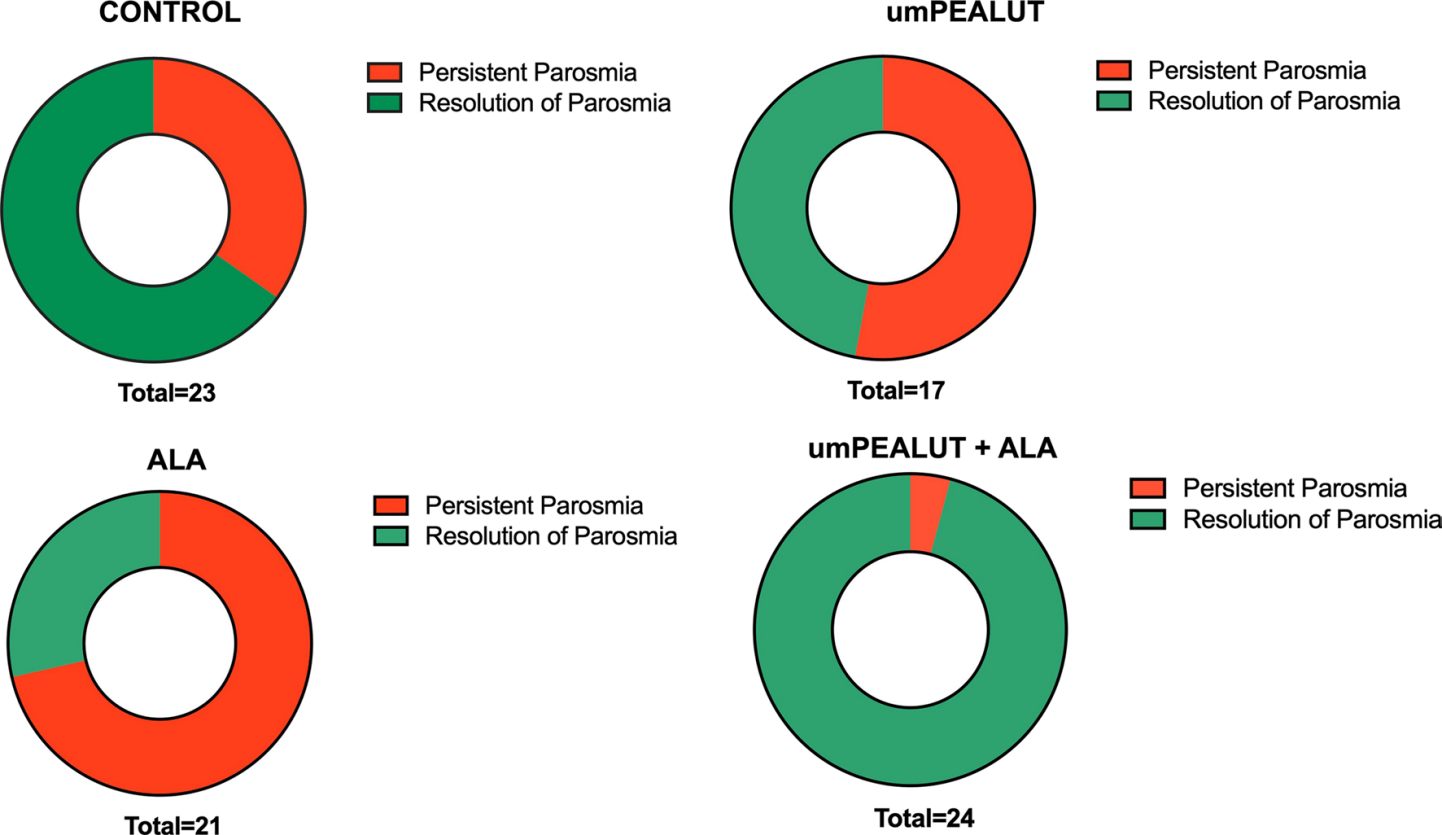
Difference in the persistence of parosmia after 6 months in the four study groups. The best recovery was obtained with the combination of umPEALUT with ALA, followed by umPEALUT alone and ALA. Olfactory training alone (control group) did not result in improvement in parosmia

Comparison of rates of resolution of parosmia showed a statistically significant increase in resolution of parosmia in the umPEALUT group compared to the ALA group (χ: p < 0.001). The combined group showed a statistically significant improvement of parosmia compared to control χ: p = 0.007, to ALA χ: p < 0.0001 and to umPEALUT χ: p = 0.001 (Fig. 4).

## Discussion

Few evidence-based treatments exist for post-viral olfactory dysfunction, and none to our knowledge have been reported for post-COVID qualitative disorders of smell. In this study, treatment with umPEALUT + OT was associated with recovery of TDI scores. The combination of umPEALUT + ALA + OT gave improvement in parosmia with less effect on TDI scores (less recovery than umPEALUT + OT) in patients affected by Long-COVID. These findings suggest that both regimens might have salutary effects for quantitative disorders of smell. In contrast, ALA + OT or OT alone were associated with little or no benefit. The difference in TDI recovery and parosmia recovery between umPEALUT and umPEALUT + ALA regimens could be related to the intrasubject variability. We did not observe significant differences in parosmia resolution between umPEALUT and control, suggesting possible benefit of ALA for parosmia. The control group had slightly higher TDI scores (better olfactory function) at the baseline, which might have favored resolution of the parosmia, given its link to quantitative smell function [2, 22], although higher TDI scores might have reduced the likelihood of improvement. Moreover, participants in the control group were, on average, affected by pOD for shorter duration than the other three groups (average 8.1 months), and these patients might therefore have a higher likelihood of spontaneous resolution of OD.

The small sample size allowed to use only two variables in the multilinear regression analyses and we analyzed TDI at the baseline and at the end of the treatment in relation to parosmia; despite an association between TDI and presence/resolution of parosmia, this association was weak in Control and umPEALUT groups and moderate in the other two groups (ALA and umPEALUT + ALA).

The differential efficacy of ALA and um-PEALUT on the quantitative versus qualitative smell disorders might reflect how these molecules affect peripheral and central components of the olfactory pathway. Li et al. [23] proposed that olfactory dysfunction arises from damage to the olfactory epithelium, olfactory bulb, and higher brain centers. Thus, the pathogenesis of smell disorders and their resolution may differ for quantitative [6] and qualitative aspects of olfaction [7]. In a position paper on COVID-19 olfactory dysfunction, Whitcroft and colleagues [20] considered the effect of SARS-CoV-2 infection on the olfactory structures and the related clinical implications. Since umPEALUT acts primarily on the brain, whereas ALA is thought to act more peripherally, the differences observed in olfactory recovery across regimens may reflect different sites of action. PEA modulates the microglial response, potentially reducing inflammation in the olfactory bulbs; the resulting environment could be more conducive to normal reconnection of the olfactory neurons with glomeruli and regrowth of the afferent olfactory pathways [7].

The experimental groups had relatively similar age, gender distribution, and severity of olfactory dysfunction at baseline, which facilitated comparisons. This similarity is helpful, since greater spontaneous recovery of olfactory losses is observed in younger patients [13]. The use of ALA combined with OT to support the recovery of lost olfactory functions caused by viral infections of the respiratory upper tract was proposed by Hummel et al. in 2002[14]; however, its efficacy with OT for COVID-19 olfactory dysfunction remains controversial. Helman et al. identified potential benefit in using ALA plus OT for treating COVID-19 smell disorders [15]. In contrast, Hopkins et al. favored use of the OT, questioning the role of alpha-lipoic acid as a standard for treating olfactory impairment caused by SARS-CoV2 infection [24].

Patients treated by ALA experienced less improvement in TDI scores than those treated with umPEALUT. We hypothesize that this finding reflects greater efficacy of umPEALUT in alleviating neuroinflammation in the superior olfactory pathway. ALA’s peripheral effect on olfactory nerves likely supports the recovery of peripheral smell losses. Although SARS-CoV-2 damages the neuroepithelium [20, 23], associated inflammation can also ascend into the olfactory bulbs through the olfactory nerve, worsening the olfactory impairment [24]. Thus, although destruction of supporting cells in the neuroepithelium is likely the primary cause of anosmia/hyposmia observed in COVID-19 patients [25, 26], inflammation of the olfactory bulbs and the central olfactory pathways likely also play a critical role in smell alterations in some COVID-19 patients [27]. The latter hypothesis is supported by tissue and immunohistochemistry studies [28], by radiological evidence [29, 30], and by the recovery of the olfactory function obtained combining anti neuroinflammation molecules with olfactory training [10–12].

Post-COVID parosmia likely represents a complex interplay of neuroepithelial and olfactory bulb damage [5, 6, 24]. Li et al. [23] proposed that quantitative disorders of smell involve (i) damage to sustentacular cells, (ii) loss of olfactory neurons, (iii) damage to horizontal basal cells with impairment in cells renewal, (iv) persistent inflammation of olfactory epithelium, (v) inflammation of the olfactory bulbs, and (vi) depletion of olfactory receptors. The onset of parosmia (qualitative disorders) can be explained with two hypotheses: the miswiring hypothesis—in which aberrant neural regeneration leads to random and incorrect axonal connection—and the incomplete neuronal regeneration hypothesis whereby correct axonal regeneration occurs but only for selected receptor types, leading to incomplete characterization and misclassification of an odor. Central contributions are also likely.

Based on current understanding of umPEALUT as a modulator of microglia that promotes a repair phenotype (M2) [31], we hypothesize that reduction of neuroinflammation can ameliorate parosmia. Reduced inflammation allows normal growth of immature neurons and facilitates the re-growth of connections between olfactory neurons and glomeruli [32]. The reduction of inflammation can support normal synaptic reconnection and prevent aberrant olfactory nerve regeneration [7]. In addition, PEA can inhibit inflammation in the olfactory bulb [23], interacting with S-protein and ACE-2 receptor [33]; this might limit the persistent inflammation of the neuro-epithelium [34, 35].

ALA’s effects on peripheral nerve could facilitate the restoration of olfactory pathways; however, the addition of ALA did not further enhance the quantitative recovery of the smell (and potentially reduced it); therefore; our results suggest a role for umPEALUT + OT in patients who are affected primarily by quantitative smell loss and umPEALUT + ALA and OT in the patients who have normal or minimal quantitative olfactory function but suffer primarily from persistent parosmia.

### Study limitation

This study has several limitations. First, the study had a small sample size (89 patients), differential attrition across groups with variation in baseline olfactory status, and limited geographical representation; therefore, results must be considered preliminary and studies with larger sample size are needed to corroborate findings. Moreover, the control group had higher TDI score at the baseline, and this could have limited the likelihood of improvement in this group. Second, adherence to the treatment regimen relied on self-report, and the study did not incorporate biomarkers, neuroimaging, or serum sampling to assess pharmacokinetics or effects of therapy on neuroinflammation. Third, the control group used OT without a placebo. Additional minor limitations included no comparator group without olfactory training, consistent with best practice of offering OT to patients with olfactory dysfunction; inherent limitations of tools for assessing parosmia, lack of data on improvement of parosmia without resolution; slight differences in the mean ages of patients across the groups; minor differences in mean months that patients were affected by olfactory loss, and prevalence of women in the combined group; although gender does not appear associated with olfactory recovery [11, 13, 36].

## Conclusions

In patients with chronic olfactory dysfunction, umPEALUT, with or without ALA, has a potential role in promoting recovery from loss of smell and parosmia. Compared to umPEALUT alone, the combination of umPEALUT with ALA more effectively reduced parosmia but was associated with lower TDI score recovery. Further studies are needed to delineate the differential effects of these therapies on damage to peripheral and central components of olfactory pathways.

## Data Availability

The data are available under resonable request to the corresponding authors.
